# Pathogenicity of a Canine-Derived Feline Panleukopenia Virus in Cats and NS1 Evolution Associated with Adaptation to Dogs

**DOI:** 10.3390/ani16071061

**Published:** 2026-03-31

**Authors:** Jiayi Wu, Qiqi Chen, Yin Zhang, Xinyuan Hu, Yongtao Li, Menghua Tang, Qingting Yu, Hua Yue, Cheng Tang, Xi Chen

**Affiliations:** 1 Key Laboratory of Animal Medicine of Sichuan Education Department, Southwest Minzu University, Chengdu 610041, China; 18631442965@163.com (J.W.); 17823202312@163.com (Q.C.); 18325029783@163.com (Y.Z.); watntyayaya@163.com (X.H.); tmh18870353465@163.com (M.T.); yhua900@163.com (H.Y.); tangcheng101@163.com (C.T.); 2 College of Veterinary Medicine, Henan Agricultural University, Zhengzhou 450046, China; yongtaole@126.com; 3 International Joint Research Center of National Animal Immunology, College of Veterinary Medicine, Henan Agricultural University, Zhengzhou 450046, China; 4 Animal Husbandry Science Institute of Ganzi Tibetan Autonomous Prefecture, Kangding 626000, China; 18990466259@163.com

**Keywords:** feline panleukopenia virus, canine-derived variant, NS1 mutations, cross-species transmission, host adaptation, attenuated virulence, viral shedding, kittens

## Abstract

Feline panleukopenia virus (FPV) is a major feline pathogen, but canine-derived FPV variants have recently been identified. Here, we compared the pathogenicity of a canine-derived FPV strain in cats with that of a lethal feline-derived FPV strain and evaluated the evolutionary significance of its NS1 mutations. Kittens infected with the canine-derived strain developed only mild, self-limiting diarrhea without fever or mortality, whereas those infected with the feline-derived strain developed severe disease and reached humane endpoints by 9 dpi. The canine-derived strain caused prolonged fecal shedding from 6 to 38 dpi but only low tissue viral loads (10^1^–10^3^ copies/g), while the feline-derived strain reached markedly higher loads (10^3^–10^6^ copies/g), particularly in the ileum, jejunum, and lungs. Viral DNA levels in the lungs, ileum, caecum, and rectum were significantly higher in the feline-derived group. Sequence analysis identified four NS1 mutations, 115I, 132L, 247Q, and 595Q, which showed stepwise evolutionary accumulation and signatures of positive selection. These findings indicate that canine-derived FPV retains infectivity in cats but exhibits attenuated pathogenicity and reduced replication fitness, highlighting NS1 as a potential determinant of host adaptation.

## Simple Summary

Cats can suffer a serious and sometimes fatal intestinal disease caused by a parvovirus that has traditionally been considered a cat virus, while a related parvovirus circulates in dogs. In recent surveillance, we found a cat-type parvovirus that was detected in dogs and carried unusual changes in a viral replication protein, raising concern about what would happen if it returned to cats. Here, we orally infected specific pathogen-free kittens with this dog-derived cat parvovirus and compared the outcome with infection by a highly virulent cat strain. Kittens exposed to the dog-derived virus developed only mild, short-lived diarrhea, did not develop fever, and survived, but they shed virus in feces for a long period, and the virus could be detected at low levels in multiple organs, indicating a body-wide infection with limited growth. In contrast, kittens infected with the virulent cat strain developed rapid, severe disease with high virus levels and died. Genetic analyses suggest that changes in the replication protein may have been favored during adaptation in dogs. These findings highlight a potential route of viral movement between dogs and cats and support continued surveillance of emerging FPV variants.

## 1. Introduction

Feline panleukopenia virus (FPV), a member of the genus Protoparvovirus within the family Parvoviridae, is a highly contagious pathogen that causes severe and often fatal disease in cats [1,2]. FPV possesses a ~5.2 kb single-stranded DNA genome encoding two non-structural proteins (NS1 and NS2) and two capsid proteins (VP1 and VP2). Among these, VP2 constitutes approximately 90% of the viral capsid and is a principal determinant of host range, tissue tropism, and antigenicity [3,4]. NS1 is indispensable for viral replication, genome packaging, and modulation of host responses [5]. It contains an origin of replication-binding region (aa 1–337), a helicase/ATP-binding region (aa 338–556), and a C-terminal transactivation domain (aa 600–667) [6]. NS1 mediates ATP hydrolysis, DNA binding and cleavage, helicase activity, transcriptional transactivation, nuclear localization, and innate immune suppression [7]. Key amino acid residues involved in ATP and DNA binding have been identified, and mutations at these sites can impair viral replication [8,9].

FPV infection primarily targets rapidly dividing cells, especially intestinal crypt epithelial cells, bone marrow progenitor cells, and lymphoid tissues, including the thymus [10,11]. As a result, infected cats typically develop acute enteritis, vomiting, diarrhea, leukopenia, lymphoid depletion, and immunosuppression, with young kittens often showing the most severe clinical outcomes [12,13]. These pathogenic features also provide an important framework for understanding how viral genetic variation may contribute to altered host range, virulence, and cross-species transmission [14].

Cross-species transmission is a defining feature of parvoviruses. The emergence of canine parvovirus type 2 (CPV-2) in the late 1970s exemplifies this process, as CPV-2 is thought to have arisen from FPV or a closely related virus through adaptive mutations in VP2 [4]. Several VP2 substitutions, including K80R, K93N, V103A, D323N, N564S, and A568G, have been widely used to distinguish CPV-2 from FPV [4,15]. Among these, K93N and V103A were critical for enabling binding to the canine transferrin receptor, thereby facilitating host switching [16]. The prototype CPV-2 strain initially exhibited low pathogenicity in dogs and lost the ability to infect cats [17,18], but subsequent variants, including CPV-2a and its derivatives, rapidly adapted, showing increased virulence in dogs and regained feline infectivity [4,19]. These observations raise concern that continued genetic diversification of parvoviruses may drive further changes in virulence and adaptation to new hosts.

During molecular epidemiological surveillance of diarrheic dogs in Southwest China (2019–2021), our group identified a novel FPV strain circulating in dogs and confirmed its ability to infect canines [15]. Genome sequencing showed that this virus retained typical FPV-like VP2 features but carried unique amino acid mutations in NS1 (V115I, V132L, H247Q, and H595Q) [15]. Historically, FPV was not considered a canine pathogen. However, in 2018, Pakistani researchers first identified three partial VP2 fragments of FPV from 40 canine diarrheal samples collected during 2014–2015, with key amino acid residues consistent with FPV [20]. Subsequent studies from Thailand, Vietnam, China, and Italy also detected FPV in canine fecal samples through partial or complete VP2 sequence analyses, suggesting possible FPV infection in dogs [21,22,23,24]. Notably, Italian researchers amplified two FPV genomes from dogs in Italy and Egypt [24], and their VP2 sequences were identical to the canine-derived FPV strain isolated by our group. These strains also shared NS1 mutations at residues 247 and 595, as well as a unique mutation at residue 95 [24]. Together, these findings suggest the emergence of canine-adapted FPV variants in which NS1, rather than VP2, may contribute to cross-species transmission. To date, most studies on parvoviral host adaptation have focused on VP-mediated receptor interactions, whereas the potential role of NS mutations in host range and virulence remains largely unexplored.

However, the infectivity and pathogenicity of such canine-derived FPV strains in cats remain unknown. As cats are the natural hosts of FPV, clarifying their susceptibility is important for understanding the biological consequences of viral adaptation in dogs. Here, we evaluated the pathogenicity of a canine-derived FPV strain in cats and analyzed the evolutionary characteristics of NS1, providing insight into the potential association of NS1 evolution with adaptation to dogs and underscoring the need to monitor emerging variants.

## 2. Materials and Methods

### 2.1. Virus and Animals

The canine-derived FPV strain (GenBank accession no. MZ913317) and a reference feline-derived FPV strain (GenBank accession no. OL547737) were previously identified from diarrheic dogs and cats in Southwest China and genetically characterized [3,15]. The latter was previously confirmed to be highly lethal in kittens [25]. Viral stocks were propagated in Crandell–Rees feline kidney (CRFK) cells maintained in our laboratory, and viral titers were determined by the median tissue culture infectious dose (TCID_50_) assay prior to animal inoculation.

Eight domestic kittens (2 months old) were screened before the experiment. Anal and oral swabs were collected and tested by PCR, confirming that all animals were negative for FPV, CPV-2, feline chaphamaparvovirus, feline coronavirus, feline calicivirus, and feline herpesvirus. The kittens were housed in the animal facility for one week of acclimatization and health monitoring, during which their mental state, appetite, and rectal temperature were recorded daily.

### 2.2. Ethical Statement

All animal procedures were reviewed and approved by the Institutional Animal Care and Use Committee of Southwest Minzu University (approval no. SMU-202501089) and were conducted in accordance with national and institutional guidelines for the care and use of laboratory animals. Animals were monitored at least twice daily throughout the experiment. Humane endpoints were predefined before the study. Kittens were considered severely moribund if they showed persistent recumbency or inability to stand, inability to eat or drink, marked lethargy or unresponsiveness, severe dehydration associated with continuous vomiting or diarrhea, or other signs of irreversible clinical deterioration. Animals meeting these criteria were promptly euthanized for humane reasons.

### 2.3. Experimental Infection

Eight kittens were randomly assigned into three groups: (i) canine-derived FPV infection group (*n* = 4), of which 2 were designated for tissue collection and histopathological analysis and 2 for clinical monitoring; (ii) feline-derived FPV infection group (*n* = 2); and (iii) un-infected controls (*n* = 2). Infected groups received a total dose of 10^5^ TCID_50_ of the respective virus in 1 mL of PBS by oral inoculation, while controls received 1 mL of sterile PBS. Each group was housed separately under isolation conditions. Because the feline-derived FPV strain had already been shown to be highly lethal in kittens in our previous work [25], the feline-derived infection group and the uninfected control group were limited to the minimum number required to confirm the expected outcomes, in accordance with the approved animal protocol and the 3R principle (Replacement, Reduction, and Refinement). By contrast, more animals were assigned to the canine-derived FPV group, which was the main focus of this study, to allow both longitudinal clinical follow-up and tissue collection for pathological analysis while minimizing overall animal use.

### 2.4. Clinical Monitoring

Following inoculation, kittens were observed daily for clinical signs including activity, appetite, vomiting, and stool consistency, and diarrhea severity was evaluated based on stool consistency. Stool consistency was classified as no abnormality (normal formed stool), mild (soft or loose stool), moderate (semi-liquid stool), or severe (watery diarrhea). Rectal temperatures were recorded every 24 h. Oral and rectal swabs were collected daily to monitor viral shedding.

### 2.5. Hematological Analysis

Blood samples were collected from cats in each group during the experimental period and submitted to the Animal Hospital of Southwest Minzu University for routine hematological examination. Hematological parameters, including white blood cell (WBC), lymphocyte (LYM), and neutrophil (NEU) counts, were analyzed to evaluate hematological changes associated with FPV infection.

### 2.6. Sample Collection and Histopathology

Based on the clinical manifestations observed after viral infection, the euthanasia time point for comparative histopathology was defined as the moment when kittens in the feline-derived FPV group became severely moribund. At this time, the two moribund kittens in the feline-derived FPV group were euthanized, and, in parallel, two of the four kittens in the canine-derived FPV group were randomly selected for humane euthanasia and tissue collection, while the remaining two kittens in the canine-derived FPV group continued to be monitored for clinical signs. Tissues including the thymus, lungs, heart, liver, duodenum, jejunum, ileum, caecum, colon, and rectum were collected for histopathological examination and viral distribution analysis. Representative tissues exhibiting marked pathological differences between groups were fixed in 10% neutral-buffered formalin, paraffin-embedded, sectioned, and stained with hematoxylin and eosin (H&E).

### 2.7. Viral Load and Shedding Analysis

DNA was extracted from fecal and oral swabs, as well as from homogenized tissue samples, using a commercial genomic DNA extraction kit according to the manufacturer’s instructions (Aidlab Biotech, Ltd., Beijing, China). Viral loads were determined by absolute quantitative PCR (qPCR) targeting the VP2 gene as previously described [26], and genome copy numbers were calculated according to the original published method. Differences in viral DNA loads in swab and tissue samples were analyzed using GraphPad Prism (version 10.0.0) with two-way analysis of variance (ANOVA) followed by Sidak’s multiple-comparisons test. Differences were considered statistically significant at *p* < 0.05. In the figures, significance levels are indicated as follows: *p* < 0.05 (*), *p* < 0.01 (**), *p* < 0.001 (***), and *p* < 0.0001 (****).

### 2.8. Evolutionary Analysis of NS1

A dataset of 257 complete FPV NS1 sequences available in GenBank up to August 2025 was assembled. Multiple sequence alignments were performed using MEGA X (version 10.0.5) to compare sequence variation and identify characteristic amino acid mutations in NS1. The spatiotemporal distribution of the NS1 mutations V115I, V132L, H247Q, and H595Q was analyzed to evaluate their evolutionary occurrence patterns across different regions and time periods. Sequence logos were generated using WebLogo (version 3.9.0; weblogo.berkeley.edu) to assess residue conservation and variability at key NS1 sites. A heatmap was further constructed in Python (version 3.13.5) using the matplotlib and seaborn libraries to visualize the distribution of these mutations among the analyzed strains.

### 2.9. NS1 Selection Pressure Analysis

Selection pressure analyses of the NS1 coding sequences were performed using the HyPhy package (version 2.5 series) on the HyPhy online platform. Overall synonymous (dS) and nonsynonymous (dN) substitution rates were estimated using the Nei–Gojobori method with Jukes–Cantor correction. In addition, pairwise comparisons among representative NS1 sequences were conducted to assess dN/dS patterns associated with the mutation-bearing sequence combinations identified in the canine-derived FPV lineage. Codons showing elevated nonsynonymous substitution signals were considered candidate sites potentially associated with adaptive evolution, and site-level selection patterns were further explored within the HyPhy framework.

### 2.10. 3D Modeling of NS1 Protein Structure

To evaluate the structural impact of the NS1 mutations in the canine-derived FPV strain, wild-type and mutant NS1 amino acid sequences from the canine-derived strain (GenBank accession no. MZ913317) and a prototype FPV reference strain (GenBank accession no. X55115, 1990) were subjected to homology modeling using the SWISS-MODEL server (https://swissmodel.expasy.org/interactive) (accessed on 20 May 2025). Homology models of FPV NS1 were generated using the closest minute virus of mice-related NS1 structure (PDB ID: 4r94, chain A; 64.96% sequence identity) as a template, and the resulting 3D models were visualized and compared in UCSF Chimera (version 1.1.0) to evaluate conformational changes associated with these mutations.

## 3. Results

### 3.1. Clinical Manifestations

**Figure 1:**
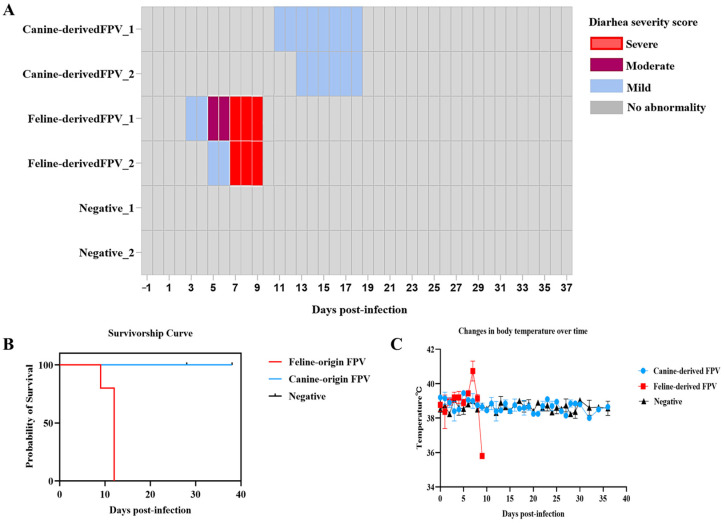
Diarrhea progression, survival, and body temperature changes in kittens following experimental infection with FPV. (**A**) Heatmap showing diarrhea progression in kittens inoculated with canine-derived FPV, feline−derived FPV, or mock control. Diarrhea severity was assessed based on stool consistency as follows: no abnormality, normal formed stool; mild, soft or loose stool; moderate, semi-liquid stool; severe, watery diarrhea. Gray indicates no abnormality, light blue indicates mild diarrhea, purple indicates moderate diarrhea, red indicates severe diarrhea. (**B**) Survival curves of kittens inoculated with feline-derived FPV (red), canine-derived FPV (blue), or negative control (gray). (**C**) Body temperature changes in kittens inoculated with the feline-derived FPV (red), canine-derived FPV (blue), or negative control (gray).

Following experimental inoculation, kittens in the canine-derived FPV group remained clinically normal until 11 days post-infection (dpi), when one kitten first developed mild diarrhea. By 11 dpi, both kittens in this group displayed mild diarrheal symptoms, which persisted until 17 dpi before gradually resolving (Figure 1A). No severe clinical deterioration was observed in these animals throughout the study period. In contrast, kittens infected with the feline-derived FPV strain developed earlier and more severe gastrointestinal and systemic signs. Mild diarrhea was first observed at 3 dpi and progressed rapidly, reaching moderate to severe watery diarrhea by 5–9 dpi (Figure 1A). In addition to diarrhea, both kittens showed lethargy, reduced appetite, recurrent vomiting, and progressive emaciation. By 9 dpi, both animals showed severe clinical deterioration, including complete anorexia, marked lethargy, progressive emaciation, and persistent vomiting and diarrhea, and had reached the predefined humane endpoints. They were therefore euthanized by intravenous administration of pentobarbital sodium (≥100 mg/kg) in accordance with institutional ethical guidelines. For ethical and experimental consistency, the two kittens in the canine-derived FPV group were euthanized at the corresponding terminal time point despite the absence of life-threatening disease. No clinical abnormalities were detected in the negative control group throughout the observation period. The survival outcomes of the three groups are shown in Figure 1B. Collectively, these findings indicate that, compared with the feline-derived FPV strain, the canine-derived FPV strain induced only mild and non-lethal diarrhea, whereas the feline-derived FPV strain caused rapidly progressive and fatal disease.

### 3.2. Body Temperature Monitoring

Throughout the observation period, kittens inoculated with the canine-derived FPV strain maintained body temperatures within the normal physiological range (38.0–39.5 °C), exhibiting only minor fluctuations until the end of viral shedding (Figure 1C). In contrast, kittens challenged with the feline-derived FPV strain displayed a markedly different pattern. Between 5 and 9 dpi, pronounced fluctuations were observed, characterized by febrile peaks followed by a rapid decline. Notably, body temperatures in affected kittens fell sharply to approximately 35 °C immediately prior to death, with mortality occurring by 9 dpi. These results indicate that feline-derived FPV induces acute and severe disruptions in thermoregulation, whereas the canine-derived FPV strain causes only mild and transient effects, with temperatures remaining within the normal range (Figure 1C).

### 3.3. Viral Shedding

**Figure 2:**
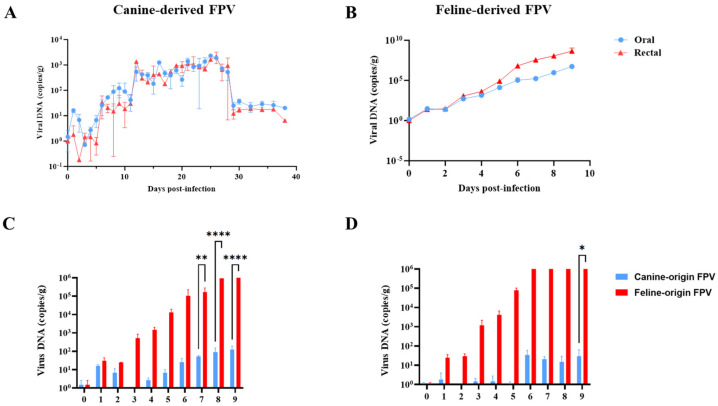
Viral shedding dynamics in kittens experimentally infected with canine-derived FPV or feline-derived FPV. (**A**) Temporal trends of oral and fecal viral shedding in kittens infected with canine-derived FPV. (**B**) Temporal trends of oral and fecal viral shedding in kittens infected with feline-derived FPV. (**C**) Comparative analysis of oral viral shedding between the two groups during the first 9 days post-infection. (**D**) Comparative analysis of fecal viral shedding between the two groups during the first 9 days post-infection. In the figures, significance levels are indicated as follows: *p* < 0.05 (*), *p* < 0.01 (**), and *p* < 0.0001 (****).

Kittens inoculated with the canine-derived FPV strain began shedding virus at 6 dpi, with viral loads gradually increasing, peaking at 26 dpi, and ceasing by 38 dpi (Figure 2A). By contrast, the feline-derived FPV strain produced a rapid rise in viral shedding that remained at relatively high levels until death (Figure 2B). Although the canine-derived FPV strain was shed for a longer duration, its overall shedding levels were substantially lower than those of the feline-derived FPV strain. To further compare shedding kinetics between the two infected groups, statistical analysis was restricted to the shared observation period (0–9 dpi). Oral viral shedding was significantly higher in the feline-derived FPV group at 7, 8, and 9 dpi (*p* < 0.001), while fecal viral shedding was significantly higher at 9 dpi (*p* < 0.05) (Figure 2C,D). Together, these results demonstrate that the feline-derived FPV strain exhibited more robust viral replication and shedding than the canine-derived FPV strain.

### 3.4. Hematological Changes in Cats After FPV Infection

Given the clear differences in clinical progression among the three groups, hematological analysis was performed at 7 dpi, when kittens in the feline-derived FPV group had already developed advanced clinical disease. At this time point, clear hematological differences were observed among the three groups. Cats in the feline-derived FPV group showed marked decreases in WBC, LYM, and NEU counts, consistent with the typical leukopenia and lymphopenia associated with FPV infection. In contrast, cats in the canine-derived FPV group exhibited relatively milder decreases in these indices, whereas no obvious hematological abnormalities were detected in the negative control group. The detailed data are summarized in Table 1.

### 3.5. Histopathological Changes

**Figure 3:**
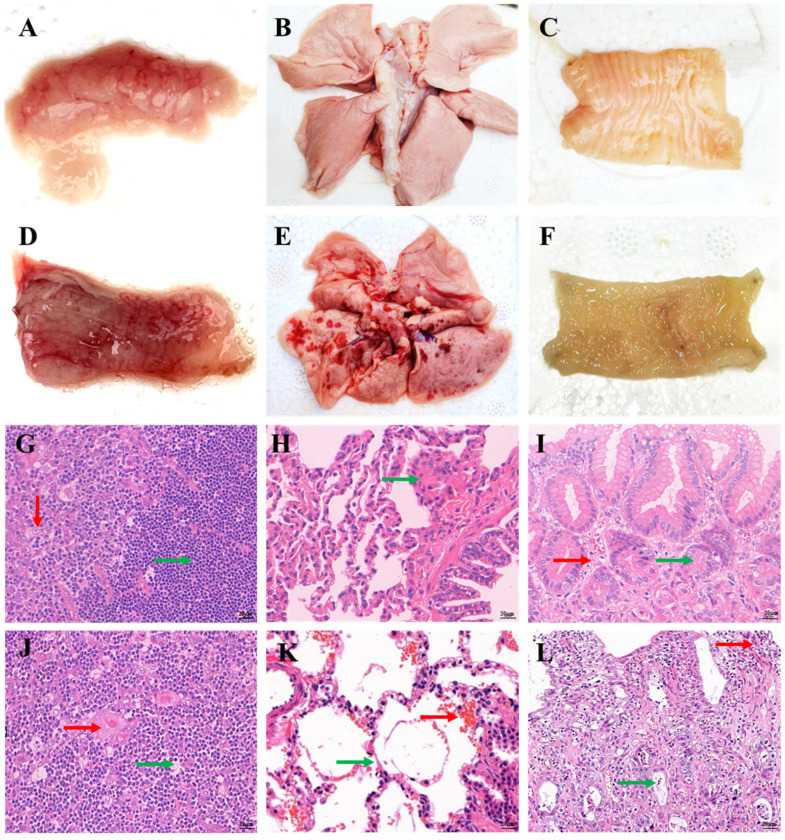
Gross and histopathological changes in kittens following experimental infection with canine-derived FPV or feline-derived FPV (400×). (**A**–**C**) Gross lesions in the thymus, lung, and intestine of kittens infected with canine-derived FPV; (**D**–**F**) Gross lesions in the thymus, lung, and intestine of kittens infected with feline-derived FPV; (**G**–**I**) Representative histopathological sections of the thymus, lung, and intestine from kittens infected with canine-derived FPV; (**J**–**L**) Representative histopathological sections of the thymus, lung, and intestine from kittens infected with feline-derived FPV.

In the canine-derived FPV group, gross lesions were largely absent, except for mild thymic hemorrhage (Figure 3A–C). By contrast, kittens in the feline-derived FPV group developed severe gross lesions in multiple tissues, most prominently in the thymus, lungs, and intestines, including thymic atrophy with hemorrhage, acute hemorrhagic pneumonia with pulmonary edema, and necrotizing enteritis of the intestines (Figure 3D–F).

Histopathological examination further demonstrated marked differences between the two infected groups. In the canine-derived FPV group, lesions were relatively mild, including thymic architectural disruption with lymphocyte necrosis, mild alveolar wall thickening with limited inflammatory infiltration in the lungs, and rectal tissues that remained largely intact with only mild mucosal edema (Figure 3G–I). In contrast, the feline-derived FPV group exhibited more severe histopathological changes, including lymphoid depletion with hemorrhagic change in the thymus, marked alveolar hemorrhage with inflammatory changes in the lungs, and epithelial degeneration with mucosal injury in the intestine (Figure 3J–L).

### 3.6. Tissue Distribution

**Figure 4:**
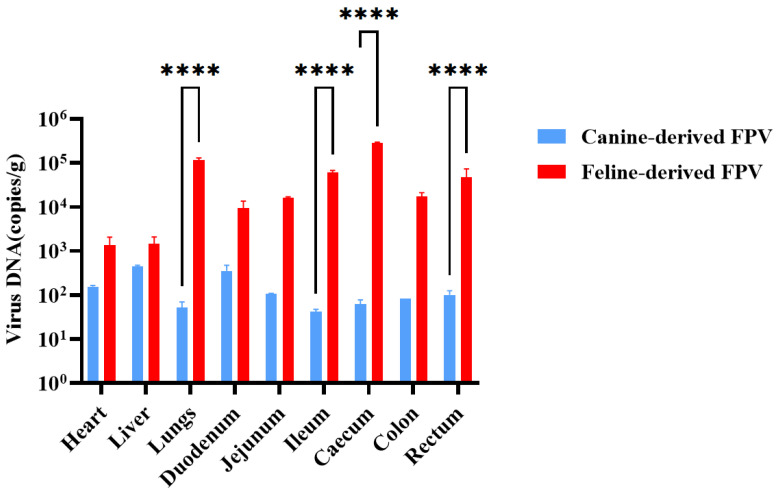
Viral distribution in tissues of kittens experimentally infected with canine-derived FPV or feline-derived FPV. *p* < 0.0001 (****).

Quantitative analysis of viral DNA copies showed that kittens infected with the canine-derived FPV strain harbored only low levels of viral DNA (10^1^–10^3^ copies/g) across all examined tissues. The highest titers were detected in the liver and duodenum. Viral DNA was detectable in both intestinal and extra-intestinal tissues, but replication remained limited. In contrast, kittens infected with the feline-derived FPV strain exhibited markedly higher viral loads (10^3^–10^6^ copies/g), with peak levels observed in the ileum, jejunum, and lungs. Statistical analysis further showed that viral DNA loads in the lungs, ileum, caecum, and rectum were significantly higher in the feline-derived FPV group than in the canine-derived FPV group (****, *p* < 0.0001) (Figure 4). These findings indicate that the canine-derived FPV strain establishes systemic infection characterized by low viral DNA loads and restricted tissue dissemination, whereas the feline-derived FPV strain shows more efficient replication, particularly in respiratory and intestinal tissues, consistent with its greater pathogenicity in cats.

### 3.7. NS1 Amino Acid Mutations and Evolutionary Dynamics

**Figure 5:**
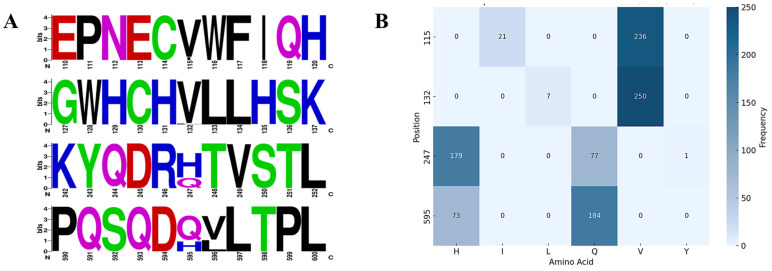
Amino acid variation at key NS1 sites of FPV. (**A**) Sequence logos illustrating amino acid conservation at positions 115, 132, 247, and 595 across FPV NS1 protein sequences. The height of each letter reflects the relative frequency of the corresponding amino acid. (**B**) Heatmap showing amino acid frequencies at the same positions.

To explore potential determinants underlying the altered host adaptation of the canine-derived FPV strain, we compared its NS1 sequence with the earliest prototype FPV reference strain deposited in GenBank and identified four amino acid mutations (115I, 132L, 247Q, and 595Q) [15]. The 115I variant was first reported in a feline isolate from Italy in 2015 (GenBank no. KX434462), whereas 132L has so far been detected only in canine-derived FPV strains described by our group (GenBank nos. MZ913314–MZ913319, OK128325). The 247Q and 595Q residues can be traced back to a jaguar isolate from China in 1986 (GenBank no. KX900570) and were first observed together in a feline isolate from China in 2007 (GenBank no. EF988660), after which FPV strains carrying the 247Q/595Q combination gradually became prevalent in cats. A feline isolate from China in 2018 was the first to simultaneously harbor 115I, 247Q, and 595Q (GenBank no. OR257444); however, no feline-derived strain has yet acquired all four mutations, suggesting that the complete NS1 motif 115I/132L/247Q/595Q remains restricted to dogs. Amino acid variation at key NS1 sites of FPV is provided in Table S1. Sequence logo and frequency analyses (Figure 5) showed that V115 and V132 are highly conserved (>90% of sequences), with 115I and 132L occurring at low frequencies (approximately 8% and 3%, respectively), mainly in canine-derived FPV. In contrast, positions 247 and 595 are more variable: 247 is dominated by H but also carries Q in about one-third of sequences and a sporadic Y, whereas 595 is predominantly Q (~70%) with H retained in the remainder. Together, these temporal and frequency patterns suggest that the canine-derived NS1 motif evolved in a stepwise manner on a background of more widespread 247Q/595Q variants and became further refined within dog-adapted lineages.

### 3.8. Selection Pressure Analysis

Selection pressure analysis of 257 FPV NS1 coding sequences revealed an overall dN/dS central tendency of approximately 0.56, indicating that the NS1 gene is predominantly subject to purifying selection, consistent with its conserved role in viral replication and transcriptional regulation. Despite this overall constraint, several codons showed elevated nonsynonymous substitution signals. Notably, residues 115, 132, 247, and 595, corresponding to the key mutations identified in the canine-derived FPV strain, were highlighted as candidate sites that may be associated with adaptive evolutionary change. Further pairwise comparisons among representative NS1 sequences showed that mutation-associated sequence combinations yielded dN/dS values approaching infinity because nonsynonymous substitutions were detected in the absence of corresponding synonymous substitutions. Although this pattern should be interpreted cautiously, it is consistent with the possibility that amino acid replacements at these positions were selectively favored during the evolution of the canine-derived FPV lineage. Detailed results for the top NS1 sites are provided in Table S2.

### 3.9. Structural Modeling of NS1 Mutations

**Figure 6:**
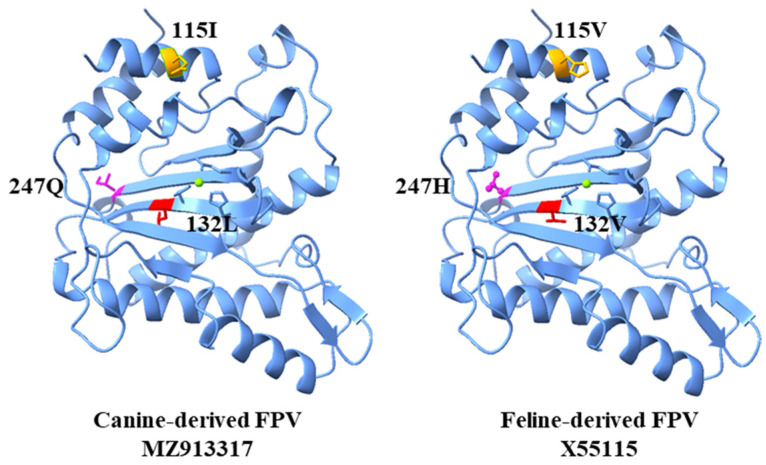
Structural comparison of canine-derived FPV and feline-derived FPV NS1 proteins.

Structural modeling revealed no detectable effect of the NS1 substitutions at positions 115, 132, and 247 on the overall protein fold or local secondary-structure organization within the modeled region (Figure 6). Because the modeling template, the murine NS1 structure (PDB ID: 4R94), spans only amino acids 6–254 of NS1, residue 595 was outside the modeled region, and its structural effect could not be assessed.

## 4. Discussion

In this study, we systematically evaluated the pathogenicity, replication, and shedding characteristics of a canine-derived FPV strain in cats and, together with NS1 evolutionary analyses, explored the potential molecular basis underlying its cross-species transmission and host adaptation. Classical experimental FPV infections in susceptible cats—whether using field strains or laboratory-adapted strains—almost invariably result in acute, severe, and often highly lethal systemic disease, and subclinical or only mildly symptomatic infections are considered rare [13,15,17]. Against this backdrop, our observation that a canine-derived FPV strain carrying the unique NS1 115I/132L/247Q/595Q combination can still establish clinical infection and persistent fecal shedding in cats while inducing only mild, self-limiting diarrhea and clearly attenuated virulence represents an unusual and informative phenotype. This low-pathogenic but still replicative state prompted us to investigate whether the distinctive NS1 mutation pattern might be linked to altered host adaptation.

Parvoviruses are well known for their capacity to cross species barriers, with the emergence of CPV-2 from an FPV-like progenitor serving as a paradigmatic example. CPV-2 acquired infectivity for dogs through a set of key mutations in the VP2 capsid protein, and the prototype CPV-2 initially exhibited low pathogenicity in dogs while losing the ability to infect cats [16,17]. Similarly, the canine-derived FPV strain analyzed in this study appears to have acquired an adaptation advantage in dogs, while in cats it produces a milder yet still replicating and shedding infection, indicating a shift in host tropism rather than complete loss of feline competence. This phenotypic shift echoes the early CPV-2 evolutionary pattern, in which adaptation to dogs was accompanied by decreased virulence in cats. However, subsequent CPV-2 variants (CPV-2a and its derivatives) rapidly adapted, showing increased virulence in dogs and regaining the capacity to infect cats [3,4,19]. Collectively, the fact that the canine-derived FPV strain can infect both dogs and cats underscores the need for vigilant surveillance at the dog–cat interface, as ongoing evolution during host adaptation could potentially enhance its fitness and pathogenicity in one or both hosts.

A limitation of the present study is the relatively small number of animals included in each group. However, the overall results from clinical observation, hematological analysis, viral shedding, tissue distribution, and histopathological examination were consistent with one another and supported the conclusion that the canine-derived FPV isolate retained infectivity in cats but exhibited attenuated pathogenicity compared with the feline-derived FPV strain. Further studies with larger cohorts will be needed to confirm these findings and to better define the contribution of NS1 variation to host adaptation.

In cats, the canine-derived FPV strain showed clearly weaker replication than the feline-derived FPV strain, with lower viral loads in multiple tissues and reduced fecal viral titers. Although viral DNA detection alone has inherent limitations, the concordant patterns of prolonged shedding and systemic distribution observed here are consistent with restricted, yet ongoing, viral activity in cats. In addition, although gross and histopathological lesions in the canine-derived FPV group were generally mild, the thymus was the only tissue showing obvious pathological change, indicating that the canine-derived FPV strain still retained the ability to affect this major lymphoid target organ of FPV infection. Hematological analysis further showed decreases in WBC, LYM, and NEU counts in the canine-derived FPV group, although these changes were milder than those in the feline-derived FPV group, suggesting that the canine-derived strain still had a measurable impact on immune cells in cats. Together, these findings indicate that, although the canine-derived FPV strain retains the capacity to infect and be shed by cats, its replication fitness and pathogenicity in the feline host are reduced. Because the VP2 protein of the canine-derived FPV strain remains FPV-like [15], it is unlikely that its attenuated pathogenicity in cats is due to impaired binding to the feline transferrin receptor. Rather, these findings suggest that the diminished virulence primarily reflects reduced replication capacity in feline tissues. Given that NS1 is the key replication-related protein in parvoviruses [5], it is reasonable to link this phenotype to the unique NS1 mutation constellation (115I/132L/247Q/595Q) and to hypothesize that these changes weaken viral replication in cats and thereby reduce adaptation to the feline host.

Our evolutionary and selection analyses further underscore the importance of these NS1 sites. Among the four focal mutations, 247Q and 595Q appeared earliest, whereas 115I and 132L arose more recently; notably, 132L is the most recent change and, so far, has been detected only in canine-derived FPV strains. In previously reported canine-derived FPV genomes from Italy, NS1 likewise carries 247Q and 595Q together with an additional mutation at residue 95 [24]. Residues 95, 115, and 132 all map to the origin-of-replication-binding region (aa 1–337) of NS1 [6], and these positions, together with 247 and 595, are under positive selection. This raises the possibility that cross-species transmission and altered host adaptation are either driven predominantly by the late-arising 132L mutation or reflect the cumulative effects of stepwise acquisition of 247Q/595Q and 115I, with 132L providing a final refinement—an issue that requires experimental testing. Collectively, these data support the view that positively selected NS1 mutations in the origin-binding region represent strong candidate determinants associated with FPV transmission from cats to dogs while reshaping viral adaptation to feline hosts. Future reverse-genetics studies introducing individual or combined NS1 mutations into a common viral backbone will be essential to dissect their precise contributions to replication fitness, virulence, and host range.

## 5. Conclusions

This study demonstrates that a canine-derived FPV strain retains the ability to infect cats, establishing systemic infection and prolonged fecal shedding, but exhibits reduced replication fitness and pathogenicity in the feline host. Evolutionary and selection analyses identified NS1 mutations as positively selected and stepwise acquired, suggesting that NS1—beyond the classical VP2 changes—may contribute to FPV cross-species transmission and host adaptation between dogs and cats. These findings highlight NS1 as an important and previously underappreciated molecular marker for FPV surveillance. Future reverse-genetics studies dissecting the individual and combined effects of these NS1 mutations will be essential to clarify their precise roles in viral replication, virulence, and host range.

## Figures and Tables

**Table 1:** Representative hematological parameters of cats in the three groups at 7 dpi.

Group	WBC (×10^9^/L)	LYM (×10^9^/L)	NEU (×10^9^/L)	Hematological Feature
Negative control	13.09	1.86	9.88	No obvious hematological abnormality
Canine-derived FPV	6.07	1.23	4.77	Relatively mild decrease
Feline-derived FPV	0.18	0.14	0.01	Marked leukopenia and lymphopenia
Reference range	2.87–17.02	0.92–6.88	2.3–10.29	—

## Data Availability

The data presented in this study are available from the corresponding author upon reasonable request.

## Supplementary Materials

The following supporting information can be downloaded at: https://www.mdpi.com/article/10.3390/ani16071061/s1, Table S1: Amino acid variation at key NS1 sites of FPV; Table S2: Selecting pressure analysis of NS1 top sites.
